# Metabolic defects induced by high-fat feeding in mice are rapidly reversed by a low-fat diet

**DOI:** 10.1186/1753-6561-6-S3-P49

**Published:** 2012-06-01

**Authors:** Nigel Turner, Simon J Leslie, Nicole L Hallahan, Gregory J Cooney

**Affiliations:** 1 Diabetes & Obesity Research Program, Garvan Institute of Medical Research, 384 Victoria St, Darlinghurst, NSW 2010, Australia

## Background

It is well established that high-fat feeding increases adiposity and impairs glucose metabolism in mice. The aim of this study was to determine the extent to which these changes are reversible if the high-fat diet (HFD) is removed.

## Materials and methods

C57BI6 mice were fed a low-fat chow diet (LFD) or a HFD (45% calories as fat) for 9 weeks and body composition, glucose tolerance and tissue triglycerides were assessed. A group of fat-fed animals were then switched to a LFD (HFD-LFD) and after 4-5 days their body composition and glucose tolerance were reassessed.

## Results

Mice fed the HFD displayed a 73% increase (P<0.01) in whole-body fat mass, an 80% elevation (P<0.01) in muscle and liver triglyceride levels and a substantial impairment in glucose tolerance compared to animals fed the LFD (area under curve during GTT: 1166 ± 76 vs. 506 ± 47 mM.min, P<0.001). The switch to a LFD resulted in a transient decrease in total caloric intake, but only a small drop in body weight (31.3 ± 0.8 vs. 30.3 ± 0.5g, pre vs. post, P=0.06). Despite the minimal change in body weight, whole-body fat mass in the HFD-LFD group was reduced almost to the level of LFD controls (12.7% vs. 14.2% by DXA, LFD vs. HFD-LFD). Consistent with the reduction in whole-body adiposity, glucose tolerance (AUC during GTT: 510 ± 49 mM.min) and muscle and liver triglycerides in the HFD-LFD animals were also restored to the level of LFD animals. Intriguingly, a separate group of mice that were pair-fed HFD to match the drop in caloric intake in the HFD-LFD group, displayed no improvements in glucose tolerance or tissue triglyceride levels.

## Conclusions

Our findings suggest that the metabolic defects induced by high-fat feeding in mice are rapidly reversible if animals are switched to a LFD.

